# Macrophage-mediated mechanisms of lung injury in the sensitization reaction to *Echinococcus granulosus*

**DOI:** 10.3389/fimmu.2024.1388120

**Published:** 2024-08-29

**Authors:** Yu-qian Li, Chun-sheng Wang, Jing-ru Zhou, Jia-ling Wang, Subi Tailaiti, Jia-ying Lin, Batesurong Bayina, Li-wei Cao, Jian-rong Ye

**Affiliations:** Department of Anesthesiology, The First Affiliated Hospital of Xinjiang Medical University, Urumqi, Xinjiang, China

**Keywords:** allergic reaction, *Echinococcus granulosus*, lung injury, macrophage, oxidative damage

## Abstract

**Objective:**

In this study, the impact of inhibiting the PI3K/AKT/NF-κB pathway on lung oxidative damage induced by *Echinococcus granulosus* cyst fluid was investigated.

**Methods:**

Twenty-four mice were randomly assigned to four groups. Three months after inoculation with hydatid cyst segments, mice in group A were treated with intraperitoneal and intratracheal saline injections; mice in group B were administered a caudal vein injection of a PI3K inhibitor, followed by cyst fluid sensitization; mice in group C received an AKT inhibitor via caudal vein, followed by cyst fluid sensitization; and mice in group D were subjected to cyst fluid sensitization without any inhibitor treatment. Cellular changes in lung tissues across all groups were evaluated, including pathological section analysis. Analysis of pulmonary tissue and serum from these mice included the assessment of PI3K/AKT/NF-κB pathway proteins, inflammatory factors, and related mRNA levels.

**Results:**

Mice in groups B and C exhibited a higher proportion of M2-type macrophages and significantly lower levels of PI3K/AKT/NF-κB pathway proteins, inflammatory factors (interleukin-6 [IL-6]/tumor necrosis factor-α [TNF-α]), and oxidative markers in lung tissues compared to mice in group D (*P <* 0.05).

**Conclusion:**

Our results in this study indicate that activation of the PI3K/AKT/NF-κB pathway contributed to an increase in the M1 macrophage phenotype, leading to enhanced secretion of peroxidases and inflammatory factors. This mechanism plays a crucial role in the oxidative and inflammatory lung damage associated with allergic reactions to *E. granulosus* cyst fluid.

## 1 Introduction

Inflammation is a complex biological process characterized by vasodilation, leukocyte migration, and tissue repair. Its primary functions include the elimination of infectious agents and other inflammatory stimuli, as well as the facilitation of tissue repair. As a fundamental defense mechanism, inflammation protects the body against infections and injuries (1). Factors such as trauma, infection, and surgery can trigger inflammatory responses, leading to beneficial effects such as combating infection. However, excessive inflammation can disrupt the balance between injury and recovery, worsening damage to target organs. Macrophages play a pivotal role throughout the inflammatory response, engaging in phagocytosis, mediator release, and reactive oxygen species (ROS) production (2). Among macrophage phenotypes, notably, the M1 subtype is instrumental in the production of the inflammasome NLRP3, a key player in pyroptosis-induced tissue damage. This process further intensifies the inflammatory response by releasing pro-inflammatory factors like interleukin-1β and other damaging factors, contributing to organ damage (3).

Patients infected with *Echinococcus granulosus* (Eg) may experience severe allergic reactions following cyst rupture during clinical procedures. These reactions can manifest as urticaria, transient rigor or fever, life-threatening bronchospasm, angioneurotic edema, and anaphylactic shock (4). Consequently, pulmonary complications pose a significant risk to patient safety during these allergic events. The degree, severity, and duration of these pulmonary complications are crucial in determining the patient’s prognosis. Furthermore, there is a marked escalation in the mortality rate in intensive care units (ICUs) with the onset of acute lung injury (5).

When an individual is infected with *E. granulosus* larvae, macrophages undergo differentiation towards a pro-inflammatory state, triggering tissue damage (6). However, specific antigenic components of the *E. granulosus* larvae, particularly the EgAgB8/1 apolipoprotein component of EgAgb, can prompt macrophages in an anti-inflammatory direction. This polarization occurs independent of interleukin-10 (IL-10) and serves to inhibit the inflammatory damage caused by factors such as tumor necrosis factor-alpha (TNF-α) (7). In humans, this phenomenon correlates with the increased expression of the pattern recognition receptor TLR4 following infection by *E. granulosus* larvae (8). The activation of TLR4 in macrophages can potentially reduce inflammatory damage (9).

The roles of activated (M1) and alternatively activated (M2) macrophages have been extensively investigated in the context of lung injury. Alveolar macrophages, residing on the lung’s inner surface, constitute 55% of pulmonary immune cells. They can polarize into two distinct phenotypes: the classically activated (M1) and the alternatively activated (M2). M1 macrophages are closely linked to a pro-inflammatory response, whereas M2 macrophages are instrumental in anti-inflammatory responses (10). During inflammation, an increase in interleukin-4 encourages macrophages to release factors such as IL-10 and stromal cell-derived factor 1 alpha (SDF-1α) that are involved in reparative functions, thus mitigating the inflammatory response’s damage to tissues and promoting recovery (11).

In conclusion, following an infestation with *E. granulosus* larvae, macrophages demonstrate dual roles: they provide anti-inflammatory protection by secreting factors that promote tissue repair while simultaneously releasing inflammatory factors that can contribute to tissue damage. In this study, the macrophage-mediated mechanisms of lung injury resulting from allergic reactions to *E. granulosus* cyst fluid were explored. Our objective was to identify the cellular processes that minimize lung tissue damage in these allergic responses, ultimately alleviating patient suffering and accelerating recovery.

## 2 Materials and methods

### 2.1 Equipment used in the experiment

The experimental setup included a micro-volume nucleic acid protein detector (SMA4000, Merinton), transfer membrane device (170-4070, Biorad), ultra-clean workbench (SW-CJ-1FD, Suzhou Antai Airtech Co., Ltd.), low-temperature refrigerated centrifuge (3K15, ThermoFisher), real-time detection instrument (7500, ABI), flow cytometer (5810R, Eppendorf), and additional necessary equipment (DXFLEX, Beckman).

### 2.2 Materials used in the experiment

Materials used in the experiments were Real Time PCR Easy™-SYBR Green I (QP-01014,FOREGRNR), PrimeScript™ IV 1st strand cDNA synthesis (6215A, TakaRa), BCA protein assay kit (BL521A, Biosharp), SDS-PAGE gel preparation kit (P1200, Solarbio), RIPA strong lysis buffer (R0010, Solarbio), phosphatase inhibitor cocktail (CW2383, Cowin), protease inhibitor (BL612A, Biosharp), ECL luminescence liquid (P10300, Biosharp), PE anti-mouse F4/80 (123109, BioLegend), PerCP/Cy5.5 anti-mouse/human CD11b (101227, BioLegend), FITC anti-mouse CDC (117305, BioLegend), Alexa Fluor 700 anti-mouse CD86 (105023, BioLegend), APC/Cyanine7 anti-mouse CD45 (157203, BioLegend), FITC anti-mouse CD206 (141704, BioLegend), MK-2206 (HY-10358, MCE), and LY294002 (HY-10108, MCE).

## 3 Experimental methods

### 3.1 Animal grouping and modeling

The study was approved by the Ethics Committee of the Experimental Animal Center of the First Affiliated Hospital of Xinjiang Medical University (IACUC-20200318-04).

Female C57/BL6 mice (4–8 weeks old, with a body weight of 18 ± 2 g), obtained from the Experimental Animal Center of the First Affiliated Hospital of Xinjiang Medical University, were used as the experimental animals.

**Figure 1:**
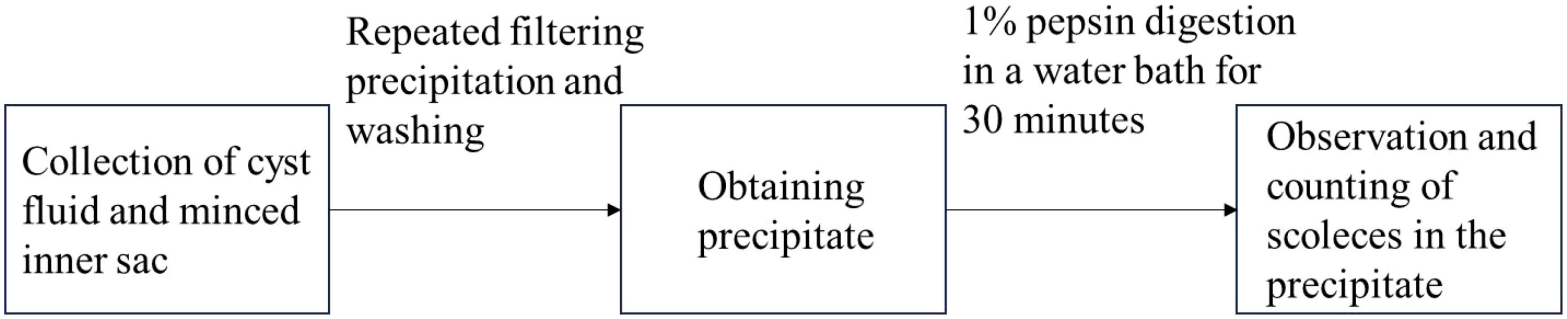
Flowchart of the Extraction of *Echinococcus Granulosus* Protoscoleces.

Crude hydatid cyst fluid was obtained from sheep livers (purchased from the Hualing Slaughterhouse in Urumqi, Xinjiang), infected with *E. granulosus* larvae, and confirmed to contain well-developed cysts. After washing and drying, the fluid with protoscoleces was centrifuged and filtered to ensure that lipopolysaccharide (LPS) levels were below 1.2 endotoxin units/mg. The protein concentration was determined to be 2815 µg/mL or higher using the BCA protein assay kit and then stored at -80°C for future use. The protoscoleces extraction process is shown in **Figure 1**.

To assess the impact of pathway inhibitors on the *E. granulosus* sensitization process, 24 mice were evenly divided into the following four groups:

The control group A (n = 6): Mice, inoculated with approximately 2000 *E. granulosus* protoscoleces and reared for three months, received intraperitoneal and intratracheal saline injections post-inoculation.

The inhibitor group B (n = 6): Similar to group A, these mice were inoculated with the protoscoleces and reared for three months. Post-inoculation, they were given a caudal vein injection of a PI3K inhibitor (5 µg/g), followed by 0.1 mL/g and 0.05 mL/g of hydatid cyst fluid intraperitoneally and intratracheally, respectively.

The inhibitor group C (n = 6): Following the same initial procedure as group B, these mice received a caudal vein injection of an AKT inhibitor (8 µg/g) and were then administered 0.1 mL/g and 0.05 mL/g of hydatid cyst fluid intraperitoneally and intratracheally.

The hydatid cyst fluid sensitization group D (n = 6): After inoculation with approximately 2000 *E. granulosus* protoscoleces and a three-month rearing period, these mice were administered 0.1 mL/g of hydatid cyst fluid intraperitoneally and 0.05 mL/g intratracheally.

Subsequently, blood samples were collected from the medial canthus vein of the mice through enucleation 15 minutes after the intraperitoneal injection of cyst fluid, and bilateral lung tissue samples were extracted for analysis.

### 3.2 Quantitative real-time RT-PCR

The serum and frozen lung tissue samples from the mice were subjected to three rounds of testing using the qRT-PCR technique. This involved analyzing the mRNA expressions of inflammatory factors and pathway proteins. To quantitatively assess gene expression levels, the threshold cycle (CT) values were normalized using glyceraldehyde 3-phosphate dehydrogenase as an internal standard. The relative expression levels of the genes were estimated using the 2^-ΔΔCT^ method.

### 3.3 PI3K/AKT/NF-κB protein detection

The detection of PI3K/AKT/NF-κB pathway proteins was performed utilizing the Western blotting method.

#### 3.3.1 Protein extraction and quantification

The protein extraction process included lysis, centrifugation, and storage (see **Table 1**). The specific method for quantification was as follows:

**Table 1:** Protein extraction process.

Step	Detailed Operational Process
Lysis	The sample was placed in a centrifuge tube, and a lysis buffer was added. It was let to sit on ice for 30 minutes, with the tube being inverted and mixed evenly every 15 minutes.
Centrifugation	Performed at 4℃ for five minutes; the supernatant solution was extracted subsequently.
Storage	Stored long-term in a -80℃ freezer

Lysis Buffer: A total of 350 μl of RIPA lysis buffer was used for the lysis process. The buffer was supplemented with PMSF to obtain a final concentration of 1 mM. A phosphatase inhibitor cocktail was added to achieve a final concentration of 1X, and a protease inhibitor was also added to a obtain final concentration of 1X.

The protein standards were completely dissolved at room temperature. Then, 20 μl of a 5 mg/mL BSA protein standard solution was diluted to 100 μl with PBS solution to achieve a final concentration of 1.0 mg/mL. The test sample was added to a microplate (diluted 10 times), and the volume was adjusted to 20 μl with PBS.

The BCA working solution was prepared by mixing reagent A and reagent B in a 50:1 volume ratio. For quantification, 200 μl of the BCA working solution was added to the microplate while ensuring thorough mixing. This was then incubated at 37°C for 30 minutes.

Note: Alternatively, the samples may be incubated for two hours at room temperature or for 30 minutes at 60°C. During protein concentration determination using the BCA method, the color of the protein solution intensifies gradually, with the colorimetric reaction accelerating as the temperature increases. In cases of low protein concentrations, it is advisable to incubate at higher temperatures or extend the incubation time as needed.

Absorbance was measured at 562 nm, and readings were recorded. Samples without BSA served as the control standard. Utilizing the BSA standard detection data, a standard curve was plotted, allowing for the calculation of protein concentration in each sample.

#### 3.3.2 Western blotting

For each sample, the loading amount was set to 20 μg, supplemented with appropriate 5 X SDS (the final concentration of β-ME was 1%), and adjusted to a 10 μl volume with buffer. Samples were boiled for 10 minutes in a 95°C water bath. Using SDS-PAGE gel, a 12% separating gel and a 5% stacking gel were prepared for electrophoresis analysis of the protein samples. Post-electrophoresis, membrane transfer, blocking, and primary antibody incubation (using one target protein/GAPDH as an internal reference) were carried out, and the samples were incubated overnight on a shaker at 4°C. The membranes were then washed three times with 1X Tris buffered saline with Tween 20 (TBST), each wash lasting 10 minutes. For secondary antibody incubation, the process was conducted at room temperature for one hour, followed by three washes with 1X TBST, each lasting 10 minutes. Finally, ECL luminescent liquid was applied for color development and imaging.

#### 3.3.3 Quantitative analysis of protein expression

The chemiluminescent signals obtained from ECL processing were analyzed using Image Lab software. These signals were converted into grayscale images to facilitate relative quantitative analysis of signal intensity.

### 3.4 Comparison of oxidative damage in lung tissue

Tissue Fixation: For fixation, one side of the C57/BL6 mouse lung tissue, ≤ 3 mm thick, was submerged in a 10% formalin solution for 24 hours, maintaining a tissue-to-fixative ratio of 1:10. Tissue Dehydration: Post-formalin immersion, the tissue was sequentially soaked in 75% ethanol for 60 minutes, 85% ethanol for 90 minutes, and finally in anhydrous ethanol I and II, each for 60 minutes. Tissue Clearing: The dehydrated lung tissue was subjected to clearing in xylene I, II, and III, each for 60 minutes. Wax Infiltration: The lung tissue was embedded using paraffin wax with a melting point of 56–60℃. Sectioning: Uniform sections, 4–6 um thick, were cut, ensuring that there were no wrinkles or knife marks. Slide Baking: The slides were typically baked in a 60℃ oven for 30 minutes. This was followed by routine gradient dewaxing for hematoxylin and eosin (HE) staining. HE Staining: Paraffin sections were subjected to hematoxylin staining for 0.5–1 minute, rinsed with tap water, differentiated in 1% hydrochloric acid alcohol briefly, washed again, and blued in 1% ammonia water for one minute. After washing under running water, they were stained with eosin for a few seconds, rinsed, and then dehydrated and mounted as per standard HE staining procedures.

### 3.5 Flow cytometry

Flow cytometry was utilized to analyze macrophage phenotypes. The specific operational procedures are detailed in **Table 2**. Analysis was performed using a flow cytometer.

**Table 2:** Flow cytometry procedure.

Step	Detailed Operational Steps
Tissue Extraction	Lung tissue from C57/BL6 mice was processed into a single cell suspension using glass slide compression, settling, and filtering steps.
Centrifugation	Centrifuged at 4℃, 300–400 g, for 4–5 minutes; the supernatant was discarded and cell viability was assessed using trypan blue staining. Cells were resuspended in an appropriate buffer to a obtain concentration of 2×10^7^ cells/mL.
Incubation	Flow cytometry antibodies (CD45, F4/80, CD11C, CD11B, and CD86 [0.5–1 μg/10^6^ cells]) were added for cell surface marker staining and incubated at room temperature for 20 minutes.
Washing	Performed twice with PBS

### 3.6 Statistical analysis

Statistical analysis was conducted using R software, version 4.3.1. The normality of data was assessed using the “shapiro.test” function, and the homogeneity of variances was checked using the “levene’s test” function in R4.3.1. Normally distributed data with homogeneous variances were expressed as the mean ± standard deviation. Intergroup comparisons were made using the “aov()” function for one-way analysis of variance (ANOVA). Significant results from the one-way ANOVA led to *post-hoc* analysis using the “TukeyHSD()” function for Tukey’s Honestly Significant Difference test, facilitating pairwise comparisons between groups. A P value of < 0.05 was deemed statistically significant.

## 4 Results

### 4.1 Flow cytometry analysis results

**Figure 2:**
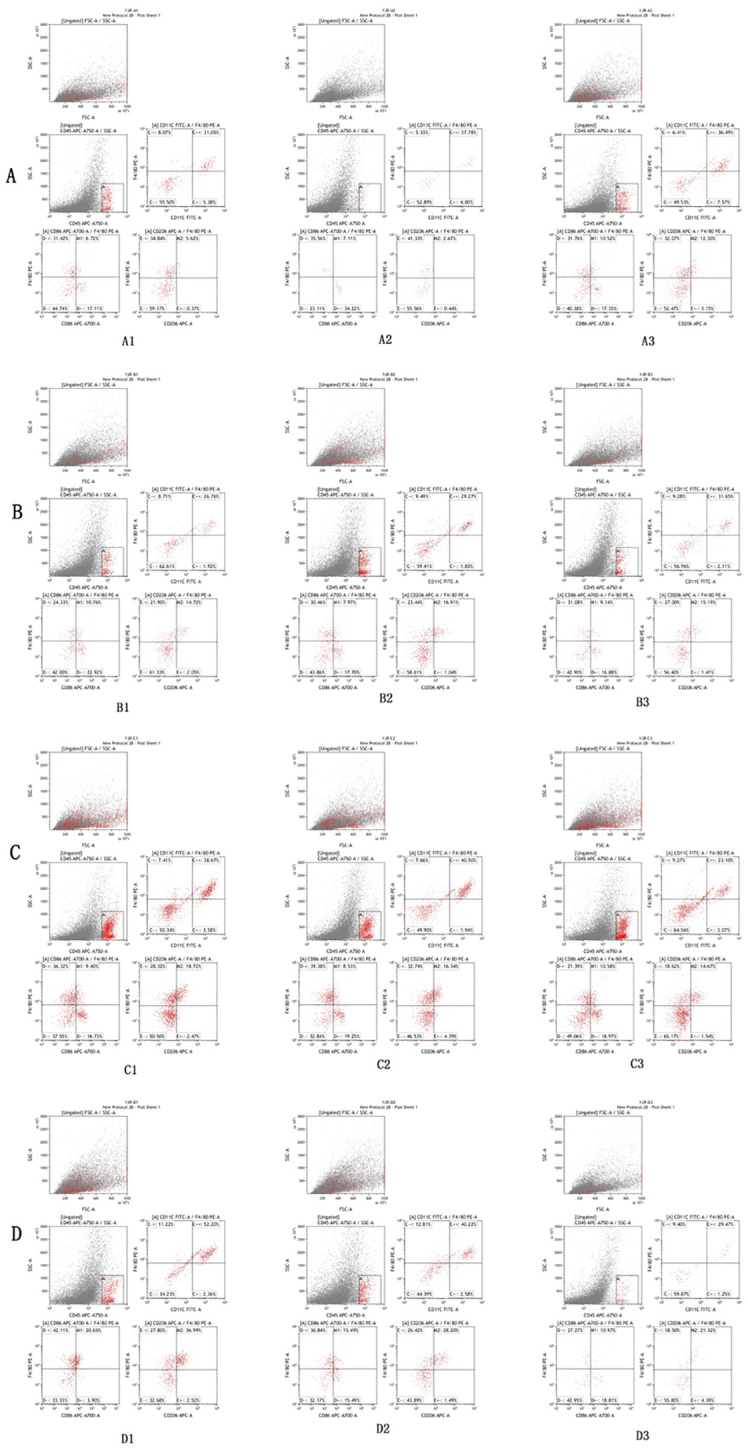
Flow cytometry analysis results of lung tissues of mice in Groups A, B, C, and D. The flow cytometry analysis reveals that the expression of M2/M1 in lung tissue macrophages in the control group **(A)** is 3.15–8.32%. Compared with Group A, a significant increase in the proportion of M2 type macrophages in lung tissue can be observed in the PI3K blocker group **(B)** and the AKT blocker group **(C)** (*P* < 0.05), while the proportion of M2 type macrophages in lung tissue is significantly decreased in the hydatid cyst fluid sensitized group **(D)** (*P* < 0.05).

Flow cytometry results, as shown in **Figure 2** and **Table 3**, were subjected to statistical scrutiny to ascertain a normal distribution and equal variances. The one-way ANOVA yielded statistically significant results (*P <* 0.05). *Post-hoc* analysis revealed a notable increase in the M1 phenotype of macrophages in the samples from the hydatid cyst fluid sensitization group D (D vs. A, *P <* 0.05), indicative of a significant rise in the pro-inflammatory phenotype. The M2 phenotype in groups B and C was significantly higher than that in groups D and A (B vs. D, *P <* 0.001; C vs. D, *P <* 0.001; B vs. A, *P <* 0.05; and C vs. A, *P <* 0.001). However, no statistically significant difference was observed between groups B and C (*P >* 0.05). This suggests a significant decrease in the M2 phenotype of macrophages in group D, with a marked increase following the administration of PI3K inhibitors (MK-2206, MCE, and HY-10358) and AKT inhibitors (LY294002, MCE, and HY-10108). The inhibitory effects of these inhibitors were statistically significant and showed similar efficacy (B vs. C, *P >* 0.05), with no notable differences between them.

**Table 3:** Flow cytometry analysis results of macrophage M2-M1 phenotype in lung tissues of mice from Groups A, B, C, and D.

Group A M2-M1 (%)	Group B M2-M1(%)	Group C M2-M1(%)	Group D M2-M1(%)
9.32	14.61	22.47	-1.1
7.81	19.86	21.5	-4.44
4.09	18.94	17.63	1.78

### 4.2 Western blot and RT-PCR results

**Figure 3:**
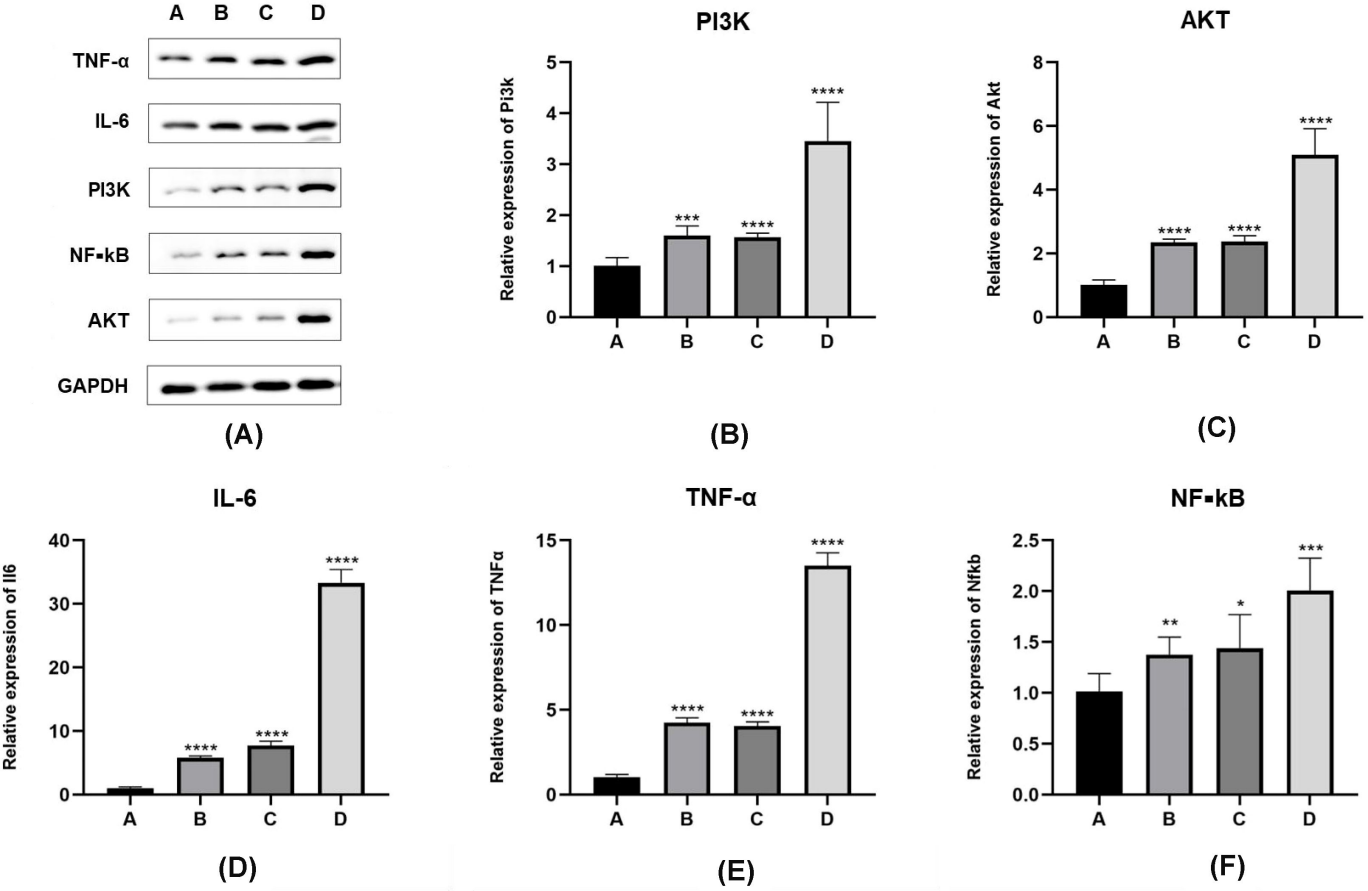
Expression levels of PI3K/Akt/NF-κB pathway proteins and inflammatory factors in *E. granulosus-sensitized* lung tissues. **(A–D)** In Western blot results, compared with inoculation control group (group A), the protein expression level of PI3K/Akt/NF-κB pathway is significantly increased in the PI3K blocker group (group B) and the AKT blocker group (group C) (*P* < 0.05). The protein expression level of PI3K/Akt/NF-κB pathway in hydatid sac fluid sensitized group is significantly increased (group D) (*P* < 0.05). Compared with groups B and C, the expression levels of the above pathway proteins in group D are significantly increased (*P* < 0.05). **(E, F)** The expression of inflammatory cytokines IL-6 and TNF-α protein is consistent with the expression trend of the PI3K/Akt/Akt/NF-κB pathway protein. Statistical significance compared to group A is indicated as follows: *: *P* < 0.05; **: *P* < 0.01; ***: *P* < 0.001; ****: *P* < 0.0001.

The variance in the expression of PI3K/AKT/NF-κB pathway proteins and inflammatory factors such as interleukin-6 and TNF-α in the lung tissues of the four mouse groups is presented in **Figure 3**. The normality of the data, shown in **Table 3**, was analyzed using the “shapiro.test” function in R4.3.1 and indicated a normal distribution with a *P* value of > 0.05. The homogeneity of variance test conducted using the “levene’s test” function in R4.3.1 also yielded a *P* value of > 0.05, confirming homogeneous variances.

The one-way ANOVA performed using the “aov()” function in R4.3.1, with subsequent *post-hoc* testing using the “TukeyHSD()” function, revealed the following:

When comparing the A, B, C, D four groups protein expression levels of PI3K, the values were 0.17 ± 0.05, 0.323 ± 0.04, 0.315 ± 0.01, 0.65 ± 0.11, mice in group D had significantly higher levels of PI3K expression compared to groups A, B, and C (D vs. A, *P <* 0.01; D vs. B, *P <* 0.01; D vs. C, *P <* 0.01). There was no significant difference between groups B and C (*P >* 0.05).

With respect to AKT protein expression values were 0.13 ± 0.05, 0.31 ± 0.09, 0.35 ± 0.11, 0.85 ± 0.11, mice in group D showed significantly higher levels than those in groups A, B, and C (D vs. A, *P <* 0.01; D vs. B, *P <* 0.01; D vs. C, *P <* 0.01), with no significant difference between groups B and C (*P >* 0.05).

Comparing the expression levels of nuclear factor kappa B (NF-κB) values were 0.19 ± 0.03, 0.382 ± 0.0357, 0.384 ± 0.0358, 0.71 ± 0.03, mice in group D had significantly higher levels than those in groups A, B, and C (D vs. A, *P <* 0.01; D vs. B, *P <* 0.01; D vs. C, *P <* 0.01). There was no statistically significant difference between groups B and C (*P >* 0.05). A separate comparison between group A and groups B and C showed significant differences (A vs. B, *P <* 0.01; A vs. C, *P <* 0.01).

Regarding the A, B, C, D four groups protein expression levels of interleukin-6 values were 0.46 ± 0.07, 0.59 ± 0.04, 0.66 ± 0.08, 0.88 ± 0.23, mice in group D had significantly higher levels than those in groups A, B, and C (D vs. A, *P <* 0.01; D vs. B, *P <* 0.01; D vs. C, *P <* 0.01). The level of interleukin-6 in group C was higher than in group A (*P* < 0.05). There was no significant difference between groups B and C (*P >* 0.05).

With respect to TNF-α values were 0.38 ± 0.04, 0.48 ± 0.02, 0.54 ± 0.08, 0.77 ± 0.11, mice in group D had significantly higher expression levels compared to groups A, B, and C (D vs. A, *P <* 0.01; D vs. B, *P <* 0.01; D vs. C, *P <* 0.05), with no significant difference between groups B and C (*P >* 0.05).

**Figure 4:**
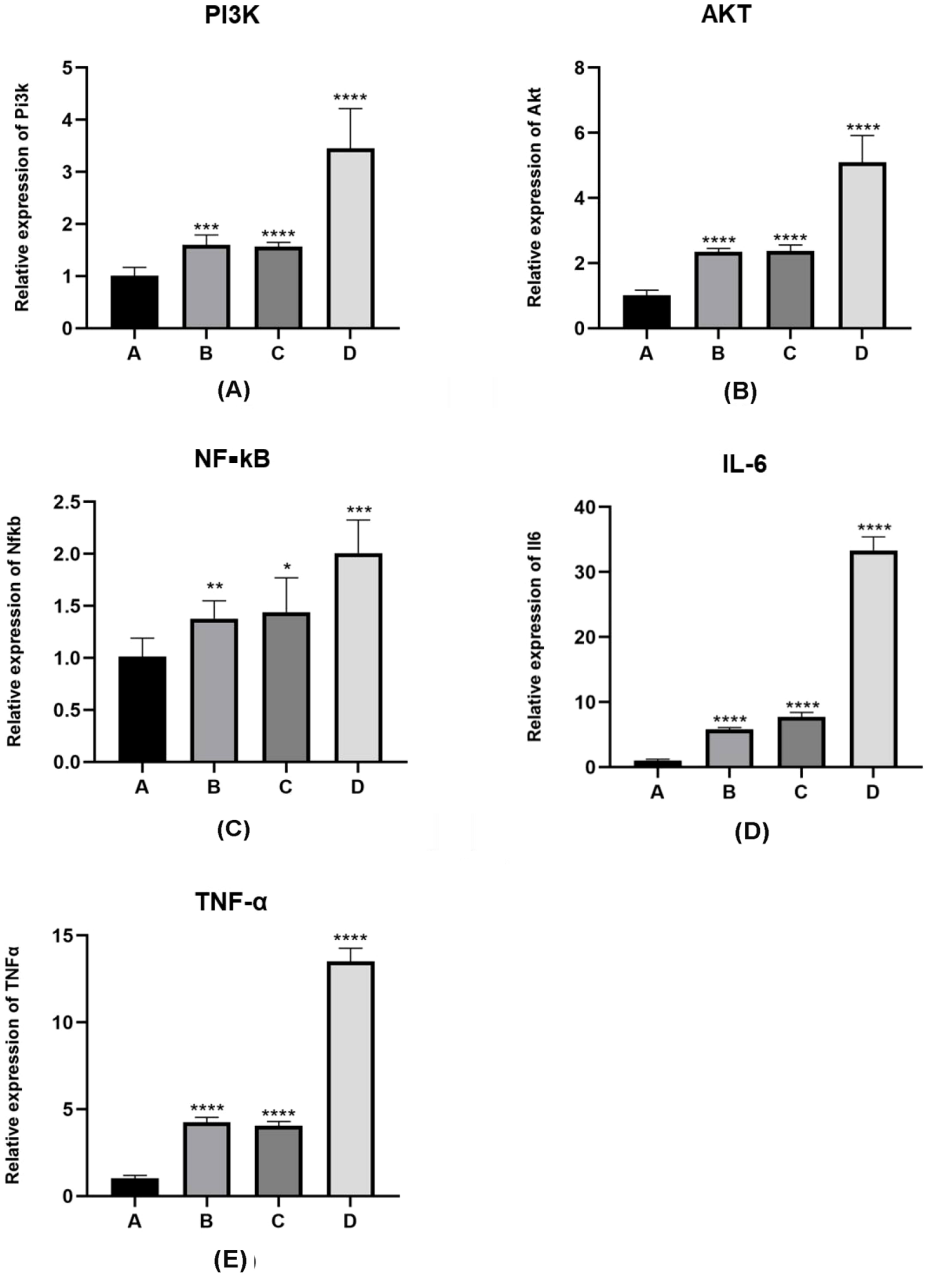
mRNA expression levels of PI3K/Akt pathway proteins and inflammatory factors in lung tissue sensitized by *E. granulosus*. **(A–C)** mRNA expression levels of PI3K/Akt/NF-κB pathway protein in the PI3K blocker group (group B) and the AKT blocker group (group C) are increased compared with the vaccination control group (group A), as per the qRT-PCR analysis results (*P* < 0.01). The mRNA levels of the PI3K/Akt/NF-κB pathway protein in the hydatid sac fluid sensitized group (group D) is significantly increased (*P* < 0.01). Compared with group B and C, the mRNA expression in group D is significantly increased (*P* < 0.01). **(D, E)** The mRNA levels of inflammatory cytokines IL-6 and TNF-α are consistent with the mRNA expression trend of the PI3K/Akt/NF-κB pathway protein. Statistical significance compared to group A is denoted as follows: *: *P* < 0.05; **: *P* < 0.01; ***: *P* < 0.001; ****: *P* < 0.0001.

The results showing the differences in mRNA expression of the PI3K/AKT/NF-κB pathway (2^-∆∆CT^ values) and inflammatory factors interleukin-6 and TNF-α (2^-∆∆CT^ values) in the lung tissues of the four groups of mice are detailed in **Figure 4**. The normality test of the data in **Table 3**, conducted using the “shapiro.test” function in R4.3.1, yielded a *P* value of > 0.05, indicating a normal distribution. The homogeneity of variance test, using the “levene’s test” function in R4.3.1, showed a *P* value of > 0.05, indicating homogeneous variances. The one-way analysis of variance (ANOVA) on the results in **Table 3** was performed using the “aov()” function in R4.3.1, with *post-hoc* testing done using the “TukeyHSD()” function.

For the A, B, C, D four groups comparison of the 2^-∆∆CT^ values for PI3K mRNA expression levels were 1.01 ± 0.17, 1.60 ± 0.19, 1.56 ± 0.08, 3.45 ± 0.76, showed that mice in group D had significantly higher levels of PI3K mRNA expression compared to those in groups A, B, and C (D vs. A, *P <* 0.05; D vs. B, *P <* 0.05; D vs. C, *P <* 0.05). In pairwise comparisons among groups A, B, and C, there was no significant difference between groups B and C, but levels in both groups B and C were higher than group A (B vs. C, *P* > 0.05; B vs. A, *P <* 0.05; C vs. A, *P <* 0.05).

For the A, B, C, D four groups of the expression of AKT mRNA expression values were 1.01 ± 0.17, 2.34 ± 0.11,2.38 ± 0.18, 5.09 ± 0.83, In the analysis of AKT mRNA expression levels, mice in group D had significantly higher levels compared to those in groups A, B, and C (D vs. A, *P* < 0.01; D vs. B, *P <* 0.05; D vs. C, *P <* 0.05). Among groups A, B, and C, there was no significant difference in AKT mRNA levels between groups B and C, but the levels in both groups B and C were higher than those in group A (B vs. C, *P* > 0.05; B vs. A, *P <* 0.05; C vs. A, *P <* 0.05).

For the A, B, C, D four groups of the expression of NF-κB mRNA values were 1.01 ± 0.18, 1.38 ± 0.17, 1.44 ± 0.33, 2.01 ± 0.32, Comparing NF-κB mRNA expression levels, mice in group D had significantly higher levels than those in groups A, B, and C (D vs. A, *P <* 0.01; D vs. B, *P <* 0.05; D vs. C, *P <* 0.05). In pairwise comparisons among groups A, B, and C, there was no significant difference between groups B and C, but NF-κB mRNA expression levels in groups B and C were higher than group A (B vs. C, *P* > 0.05; B vs. A, *P <* 0.05; C vs. A, *P <* 0.05).

For the A, B, C, D four groups of the expression of linterleukin-6 (IL-6) mRNA values were1.016 ± 0.20, 5.77 ± 0.30, 7.76 ± 0.66, 33.33 ± 2.09, mice in group D had significantly higher levels compared to groups A, B, and C (D vs. A, *P <* 0.01; D vs. B, *P <* 0.01; D vs. C, *P <* 0.01). In pairwise comparisons among groups A, B, and C, there was no significant difference between groups B and C, but IL-6 mRNA levels in both groups B and C were higher than group A (B vs. C, *P* > 0.05; B vs. A, *P <* 0.01; C vs. A, *P <* 0.01).

For the A, B, C, D four groups of the expression of TNF-α mRNA expression values were 1.01 ± 0.18,4.26 ± 0.28,4.06 ± 0.24,13.50 ± 0.77, the comparison of TNF-α mRNA expression levels revealed that mice in group D had significantly higher levels compared to those in groups A, B, and C (D vs. A, *P <* 0.01; D vs. B, *P <* 0.01; D vs. C, *P <* 0.01). Among groups A, B, and C, there was no significant difference between groups B and C, but TNF-α mRNA levels in groups B and C were higher than group A (B vs. C, *P* > 0.05; B vs. A, *P <* 0.01; C vs. A, *P <* 0.01).

### 4.3 Immunohistochemistry results

**Figure 5:**
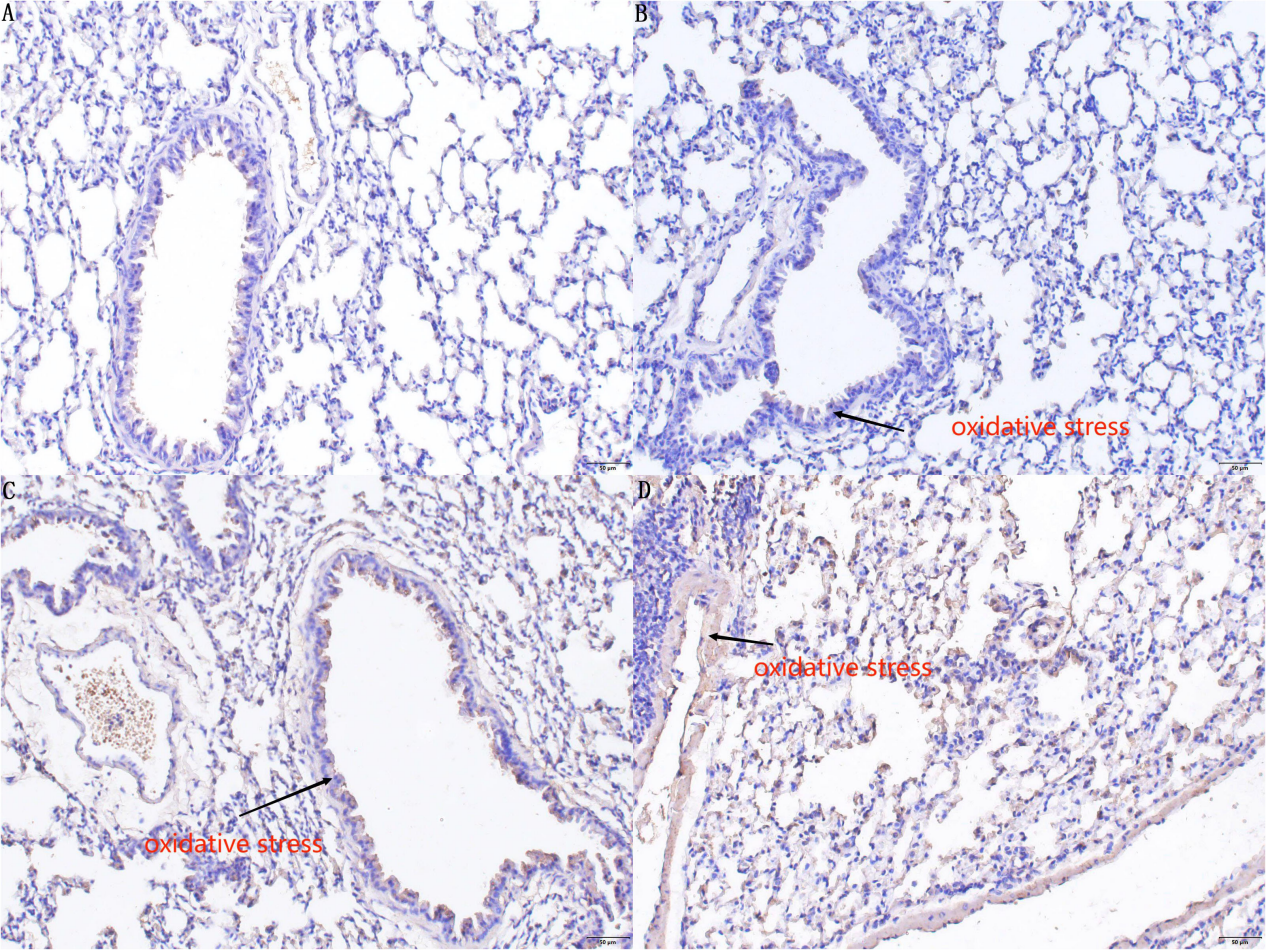
Immunohistochemistry staining results of 8-OHdG in the lung tissues of mice in groups A, B, C, and D. In the immunohistochemical results, there is no significant difference (*P* > 0.05) in the degree of DNA oxidative damage in lung tissue sensitized by *E. granulosus* in the PI3K blocker group compared to the control group **(A)**. Compared with Group A and Group B, the **(C)** AKT blocker group and **(D)** hydatid sac fluid sensitized group show more severe DNA oxidative damage in lung tissue sensitized by *E. granulosus* (*P* < 0.01). Compared with Group **(C)**, the degree of DNA oxidative damage in Group **(D)** is increased (*P* < 0.01).

Immunohistochemical analysis involved staining of 8-hydroxy-2 deoxyguanosine (8-OHdG) in the lung tissues of mice across groups A, B, C, and D, providing insights into oxidative stress levels. The quantified oxidant expression values revealed significant differences among these groups. Mice in group D demonstrated markedly higher oxidant levels in lung tissue compared to those in groups A, B, and C, with the differences being statistically significant (D vs. A: *P <* 0.01, CI 0.099–0.134; D vs. B: *P <* 0.01, CI 0.097–0.132; D vs. C: *P <* 0.01, CI 0.023–0.058). In a detailed pairwise comparison, mice in group C exhibited higher oxidant levels in lung tissue than those in group B, and mice in groups B and C showed elevated levels compared to those in group A, though the difference between groups B and A was not statistically significant (C vs. B: *P <* 0.01, CI 0.056–0.091; B vs. A: *P >* 0.05, CI 0.015–0.020; C vs. A: *P <* 0.01, CI 0.059–0.094). These findings are shown in **Figure 5**.

## 5 Discussion

Macrophages are distributed extensively in tissues throughout the body and contribute to maintaining homeostasis and resisting pathogen invasion. Depending on environmental stimuli, macrophages get polarized into distinct subtypes, namely, M1 and M2 macrophages. M2 macrophages are involved in anti-inflammatory responses and tissue repair processes (8). 8-hydroxydeoxyguanosine (8-OHdG) is one of the most commonly used markers of oxidative damage (9). An increase in the M1 phenotype of macrophages causes oxidative damage in the tissues in which they are located. For instance, in an experiment on myocardial stress response, it was found that the increase of the M1 phenotype increased the inflammatory damage and oxidative damage of cardiomyocytes, as evidenced by elevated levels of inflammatory damage markers IL-6 and TNF-α and an increase in the generation of oxygen free radicals, leading to the impairment of myocardial diastolic function (10). Similarly, in the context of intestinal inflammation, the rise in M1 macrophages compromises intestinal barrier function by promoting inflammatory responses and oxidative damage, resulting in the dysfunction of the digestive system (11).

In this study, the oxidative damage to lung tissue caused by the increase of M1 macrophages in *E. granulosus* cyst fluid during allergic reactions was examined using peroxide damage markers. Following *E. granulosus* infection, pattern recognition receptors *in vivo* in the host quickly identify the invasion, as indicated by the increase of pattern recognition receptors, specifically toll-like receptor-2 (TLR2) and toll-like receptor-2 (TLR4) (5). The increased expression of TLRs not only helps in clearing the invading exogenous substances from the body but also plays a protective role while potentially causing inflammatory damage to the body at the same time (12). The PI3K/AKT signaling pathway may induce a variety of reactions caused by oxidative damage to lung tissue and inflammatory damage (13). KEGG pathway analysis (https://www.genome.jp/kegg/) indicates that activation of pattern recognition receptors can trigger the PI3K/AKT signal pathway, resulting in the release of a variety of inflammatory cytokines, including NF-κB, TNF-α, and IL-6, which can cause acute lung injury (14).

Current research on the allergic reaction mechanisms caused by *E. granulosus* cyst fluid mainly focuses on the role of antibodies such as IgE and IgG in this allergic reaction (1), as well as the epidemiological characteristics of patients experiencing shock post-sensitization (15). This study advances the understanding of the cytological mechanisms underlying allergic reactions triggered by *E. granulosus* cyst fluid. The results demonstrate that *E. granulosus* cyst fluid can activate macrophages via the PI3K/AKT pathway, leading to the release of a large number of inflammatory factors, thereby causing tissue and cell damage. The cytological mechanism by which *E. granulosus* cyst fluid induces allergic reactions has been further refined in the study.

## 6 Conclusion

From the results of this study, we conclude that in allergic reactions triggered by *E. granulosus* cyst fluid, activation of the PI3K/AKT/NF-κB pathway plays a crucial role in the increased transformation of macrophages to the M1 phenotype. The inflammatory factors released by M1-type macrophages, such as TNF-α and interleukin-6, contribute not only to inflammatory damage but also to oxidative damage in lung tissues.

## Data Availability

The raw data supporting the conclusions of this article will be made available by the authors, without undue reservation.
